# Homology Inference of Protein-Protein Interactions via Conserved Binding Sites

**DOI:** 10.1371/journal.pone.0028896

**Published:** 2012-01-31

**Authors:** Manoj Tyagi, Ratna R. Thangudu, Dachuan Zhang, Stephen H. Bryant, Thomas Madej, Anna R. Panchenko

**Affiliations:** National Center for Biotechnology Information, National Library of Medicine, National Institutes of Health, Bethesda, Maryland, United States of America; University of South Florida College of Medicine, United States of America

## Abstract

The coverage and reliability of protein-protein interactions determined by high-throughput experiments still needs to be improved, especially for higher organisms, therefore the question persists, how interactions can be verified and predicted by computational approaches using available data on protein structural complexes. Recently we developed an approach called IBIS (Inferred Biomolecular Interaction Server) to predict and annotate protein-protein binding sites and interaction partners, which is based on the assumption that the structural location and sequence patterns of protein-protein binding sites are conserved between close homologs. In this study first we confirmed high accuracy of our method and found that its accuracy depends critically on the usage of all available data on structures of homologous complexes, compared to the approaches where only a non-redundant set of complexes is employed. Second we showed that there exists a trade-off between specificity and sensitivity if we employ in the prediction only evolutionarily conserved binding site clusters or clusters supported by only one observation (singletons). Finally we addressed the question of identifying the biologically relevant interactions using the homology inference approach and demonstrated that a large majority of crystal packing interactions can be correctly identified and filtered by our algorithm. At the same time, about half of biological interfaces that are not present in the protein crystallographic asymmetric unit can be reconstructed by IBIS from homologous complexes without the prior knowledge of crystal parameters of the query protein.

## Introduction

Protein interactions determine the outcome of most cellular processes and the analysis of protein interaction networks is crucial for understanding the mechanisms of cell functioning. The recent advances in experimental methods for identification of protein-protein interactions have provided extensive data on protein interaction networks. While for some organisms, such as yeast, the networks are close to completion and their reliability is relatively high [1], for many other organisms the protein interaction data contains a lot of false positives and the coverage still remains low. For example, it has been estimated that less than 10% of all human protein interactions have been experimentally determined [2]. Moreover, there are many self-interacting proteins in the protein interaction networks [3], but due to the ambiguity of homooligomer experimental characterization such interactions are usually poorly characterized and largely neglected in large scale network mappings.

**Figure 1:**
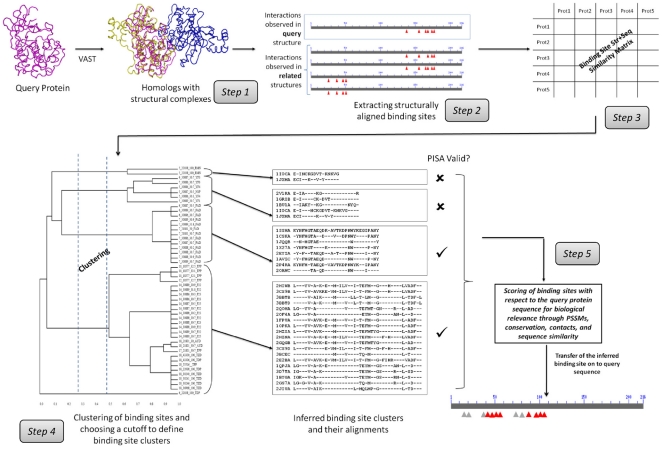
Overview of IBIS.

One way to fill this gap and provide a more reliable and comprehensive biomolecular interaction network is to employ computational methods for protein interaction prediction and verification. There are many different computational approaches to predict protein interactions; some are based on genomic context, co-evolution, co-expression or co-occurrence patterns of potentially interacting proteins and their genes [4]. Another group of methods rely on similarities between proteins with unknown interactions and homologous proteins with experimentally observed interactions [5]–[8]. It has been suggested, though, that interaction partners can be reliably inferred only for close homologs [9]–[12] and annotations transferred from one homologous protein to another may result in incorrect assignment even for close homologs if they have different binding specificities. Since binding specificity is usually determined by the structural and sequence features of protein interaction interfaces, it is essential to detect and transfer binding sites correctly. Current binding site prediction methods use either evolutionary conservation of binding site sequence motifs, information about structures of available complexes, or docking approaches if no such data is available. To verify and guide predictions based on inference, one needs to ensure similarity between unknown query protein and observed binding sites detected in homologs. Our recently developed method and server Inferred Biomolecular Interaction Server (IBIS) [13], [14] clusters similar binding sites found in homologous proteins based on the site's conservation of sequence and structure and then calculates position specific score matrices (PSSMs) from binding site alignments. Together with other measures, these PSSMs are used to rank binding sites and to gauge the biological relevance of binding sites with respect to the unknown query protein (Figure 1). Even though this server handles five different types of protein interactions (protein-protein, protein-small molecule, protein-nucleic acids, protein-peptide and protein-ion), in this work we focused only on protein-protein interactions.

In this paper we tried to assess how the homology inference approach can be used to annotate the biological partners and interfaces of protein-protein interactions even if the native complex is not present in the structural database. We try to determine which factors influence the accuracy of such an approach. First, we find that the performance of the IBIS method for predicting protein interaction partners reaches 88% sensitivity and 67% specificity while performance for prediction of binding site locations is 72% recall and 70% precision. Interestingly a considerable increase in accuracy is observed if all available data on structures of homologous complexes is used, as compared to the approach where only a non-redundant set of structural complexes is employed. Second we show that there exists a trade-off between specificity and sensitivity if we use only conserved binding site clusters or clusters supported by only one observation (singletons). Finally we address the question of predicting the biological interfaces that are not present in the PDB asymmetric unit and need to be reconstructed by applying crystallographic symmetry operations. We show that almost half of such interfaces can be reconstructed by IBIS without the prior knowledge of crystal parameters of the query protein.

## Methods

### Defining observed interactions

We used the NCBI Molecular Modeling Database (MMDB) [15] as a source of structure data on protein complexes. Protein domains are structural and functional units of proteins and many proteins evolve through domain shuffling, thereby acquiring new functions and properties. Domains over time have evolved different binding modes to interact with various binding partners. Hence to record biologically meaningful protein-protein interactions we used domains as units of interaction. We annotated domains on each protein chain using the Conserved Domain Search server (CD-Search) which provides Conserved Domain (CDD) [16] annotations for query sequences [17]. If a protein chain has multiple domains, domain-domain interaction annotations are provided separately for each domain on the query. In this study we refer to domain-domain interactions as protein-protein interactions.

A pair of interacting domains is defined if one of the domains has at least 5 residues in contact with the other. We define a residue to be in contact if there is at least one (heavy) atom of the residue within 4.0 Å of atoms of the other domain. The contact radius was chosen based on the mean number of inter-domain contacts formed by the non-redundant set of domain families. When we varied the contact radius from 2 Å to 6 Å the mean number of contacts showed a steep increase around 4 Å. The set of residues from one domain making contacts with the other domain constitutes a *“binding site”* or *“interface”*. In the current release of the Molecular Modeling Database (MMDB) [15], we found 275968 interactions from 34846 structures, 62% of which are homooligomeric (both domains or chains belong to the same CDD superfamily) and the rest represents heterooligomeric interactions. These are so-called “observed interactions”.

### Inferring interactions from homologs

To ensure the biological relevance of binding sites, they are clustered based on their sequence and structural similarity. Here are the important details concerning the main steps in the processing, as shown in Figure 1.

#### 1. Collecting homologs with observed interactions

To infer interactions by homology for a given query protein with a known structure but unknown partners and binding sites, we first collect all protein domains/chains from known complexes which are structurally similar to a query and have at least 30% sequence identity to the query (Step 1, Figure 1). Hereafter we refer to these as “homologous structure neighbors”. Structure-structure superpositions were computed using the VAST algorithm [18]. No filter was applied to remove redundant structures as there could be structures of the same protein bound to different interacting partners. We then retrieve observed interactions and binding site residues for all structure neighbors (including the query) and retain only those where at least 75% of the binding site residues are aligned to the query. At the end of this step we compile a list of all binding partners derived from structure neighbors and therefore all possible proteins predicted or inferred to interact with the query.

#### 2. Measuring binding site similarity

Next we cluster domain binding sites into groups based on their sequence and structure similarity. To construct the alignment between the structure neighbors *A* and *B* we reindex the alignment between structure neighbor *A* and the query, with the alignment between the query and structure neighbor *B* (Step 2, Figure 1). Even though there could be a “direct” alignment between neighbors *A* and *B*, we compose the alignment through the query, since neighbors *A* and *B* could be more closely related to each other than to the query and their “direct” alignment could include binding sites that are not relevant to the query. The overall similarity score between any two aligned binding sites *A = {a_1_,…,a_N_} and B = {b_1_,…,b_N_}* can be calculated by summing up over all positions *i* in the gapped alignment as follows.
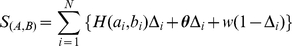
(1)where *H* is the element of the BLOSUM62 matrix, *a_i_* and *b_i_* are amino acids or gap characters in position *i* of binding sites *A* and *B*; *Δ_i_* is equal to 0 if *a_i_* or *b_i_* is a gap character and 1 otherwise; 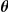 is an additional weight of “+1” added to each position (even if amino acids in the aligned positions are not very similar to each other, they are still located in the equivalent positions in two proteins and are rewarded by adding the 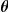 weight) and *w* is a gap penalty of “−4”. The raw score is then converted to a bit score with the statistical parameters λ and *K* previously defined in the BLOSUM scoring system [19], [20].

The similarity score is then normalized by dividing by the maximum of the bit scores when the binding sites are scored against themselves. This step serves to normalize the similarity so that the conservation scores from different interface alignments can be compared (Step 3, Figure 1).
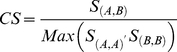
(2)


#### 3. Clustering of binding sites

The binding sites of the homologous structure neighbors are clustered using a complete-linkage clustering algorithm, which calculates the distance between two clusters as the maximum distance between their members. A distance cutoff value to define the clusters is chosen using a pseudo-free energy function from a study which maximizes the mean similarity of members within a cluster and minimizes the complexity of the description provided by cluster membership [21] (Step 4, Figure 1).
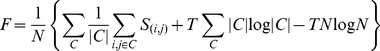
(3)where *T* is the temperature factor, *S_(i,j)_* is the similarity score between binding site *i* and binding site *j* in each cluster, *C* represents a cluster, |*C*| is the number of binding sites in the cluster *C*, and *N* is the total number of binding sites clustered. The temperature *T* constant (0.05) is chosen to correctly balance the energy-like and entropy-like terms in the function [21]. At the end of this procedure sets of binding residues (“binding sites”) from different homologs of the query protein are grouped together based on their similarity.

#### 4. Ranking of binding site clusters

All binding site clusters are ranked in terms of biological relevance and similarity to the query (Step 5, Figure 1). First, to increase our confidence that the binding site is biological and is not specific to only one protein subgroup, we check whether the same or similar binding sites reoccur in diverse protein complexes and assess their conservation within the cluster. Clusters that have more than one non-redundant protein (at a sequence identity threshold of 90%) in the cluster are called “conserved binding site” clusters. Those clusters which have only one non-redundant protein complex are considered “singletons” and usually correspond to either lineage specific binding modes or those cases where not enough evidence is obtained about their conservation. Singletons are not assigned any score and are ranked at the bottom of the ranking list. Positional conservation in the binding site alignment is calculated using the Shannon entropy measure with the Henikoff-Henikoff sequence weights (*Z_conserv_*).

Second, since the larger interfaces are more likely to be biological, the ranking score also includes the term corresponding to the number of interfacial contacts averaged over all homologous complexes (*Z_contact_*). Another term in the ranking score accounts for the relevance of a given binding site cluster to the query. A position specific score matrix (PSSM) is constructed based on the binding site alignment using the implicit pseudo-count method [22]. The aligned binding site region of the query protein is scored against the PSSM and a sequence-PSSM score is calculated (*Z_PSSM_*). A higher sequence-PSSM score implies a higher probability of this site being biologically relevant for annotating the given query. In addition we calculate the average sequence identity between the query and all cluster members over the whole structure-structure alignment (not just binding sites) to estimate the evolutionary distance between the query protein and the group of homologous structure neighbors (*Z_pcnt_*).

All components of the ranking score (i.e. PSSM, conservation, contact number, and percent identity of the alignment) are converted to Z-scores and their weighted combination is used where weights were determined empirically.
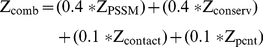
(4)


#### 5. Validation of interactions using the PISA algorithm

Interfaces present in PDB asymmetric units (ASU) are validated using the PISA (Protein Interfaces, Surfaces, and Assemblies server) algorithm [23] which is considered to be one of the best methods for identifying biologically relevant interfaces present in crystal structures [24]. PISA is an automated method for detecting macromolecular assemblies based on the analysis of interfaces and stability of assemblies reported in crystal structures. PISA uses chemical thermodynamics calculations to compute a set of macromolecular assemblies, which are expected to be stable in solution and presumed to represent the biological form of a protein in the cell.

### Evaluation of prediction accuracy

The sensitivity, specificity, precision and recall were estimated as follows. The sensitivity and error rates were estimated based on the number of true positives (correctly predicted actual pairwise interactions or binding site residues) and false positives (incorrectly predicted actual pairwise interactions or binding site residues). Sensitivity or Recall (TP/(TP+FN)) was defined as the number of true positives (TP) found divided by the overall sum of true positives in the test set. Error rate (FP/(FP+TN)) or specificity (one minus error rate) was estimated as the number of false positives (FP) divided by the sum of false positives and true negatives (TN, nonbiological interactions or binding site residues). Precision (TP/(TP+FP)) was also calculated to compare the performance of IBIS to other methods.

## Results

### Handling crystal packing interactions

To filter out fallacious interactions we have used the PISA algorithm. We regard the interactions occurring in an assembly predicted to be stable by PISA as biologically relevant, and the others occurring in the ASU but not validated by PISA as crystal packing interactions. After processing all 34846 protein X-ray complex structures having at least one observed inter-chain protein-protein interaction in the asymmetric unit (ASU), we found that 24089 (69%) of the structures are annotated to be multimeric, 6272 (18%) of structures are predicted to be monomers according to PISA and the remaining 4529 (13%) could not be processed due to various reasons such as incomplete X-ray data, for example (Figure S1). The distribution of the number of chains in the asymmetric unit which are predicted to be monomers by PISA (out of 6272 structures) is shown in Figure S2.

### Reconstructing biounits by homology inference

**Figure 2:**
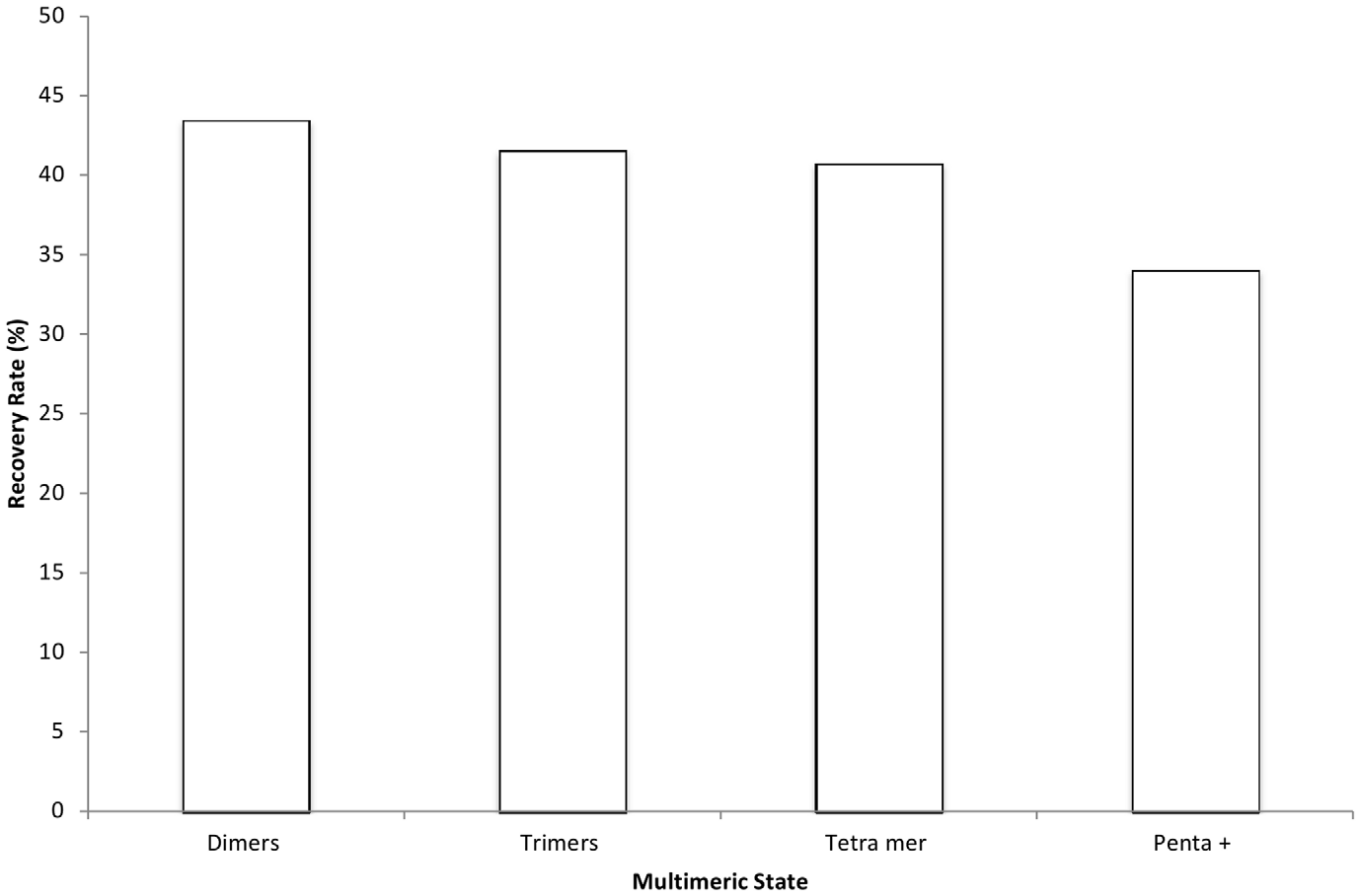
Reconstructing biounits by homology inference. Recovery of those homooligomeric interfaces by IBIS which can only be produced by applying crystallographic symmetry operations to PDB ASU. Recovery rate is calculated as a number of binding site residues identified by both PISA and IBIS divided by the number of binding site residues identified by PISA by applying crystallographic symmetry operations.

It has been noted previously that correct assignment of biological units in protein complexes can add more domain-domain interfaces beyond those that are seen in the PDB asymmetric units [25]. In these cases, transformation matrices should be used to generate the biologically relevant biounit from the asymmetric unit. We found 6000 single chain entries in the PDB ASUs that are predicted to be multimeric proteins by PISA and the majority of these are dimers as can be seen from Figure S3. Another approach to predict and verify the correct oligomeric state or biounit of a protein is to infer the oligomeric state from its close homologs. This task can be achieved within the IBIS framework (Figure 1). We used all chains from 14744 structures containing at least one interface generated by applying crystallographic symmetry operations (according to PISA) as queries in IBIS. Then we collected all binding sites annotated by the IBIS algorithm and compared them with the interface generated by PISA. True positives were defined as those cases where more than half of the IBIS binding site residues overlapped with PISA interfaces. As can be seen from Figure 2, even though these interfaces were not present in the PDB ASU, more than 40% of homodimeric and higher order oligomeric novel interfaces can be reproduced by homologs using IBIS.

### Evaluation of IBIS performance to predict protein-protein interactions

We evaluated the accuracy of IBIS to predict protein-protein binding sites from three different perspectives. First we compared IBIS annotated sites with manually curated CDD annotated sites. The definition of false positives for protein-protein interaction predictions is rather ambiguous and none of the available test sets of true interactions can capture all possible biologically relevant interactions for a given protein. Therefore we evaluated separately the rate of true positives and false positives using two test sets: the test set of crystal packing pairwise interactions to evaluate the false positive rate (specificity) and a set of biological interactions to evaluate the true positive rate (sensitivity). Finally we performed comparisons with other available methods on the test sets reported previously. In this case the test sets provided the information not only on pairwise interactions but also on the locations of binding sites.

#### Comparison with CDD annotated binding sites

**Figure 3:**
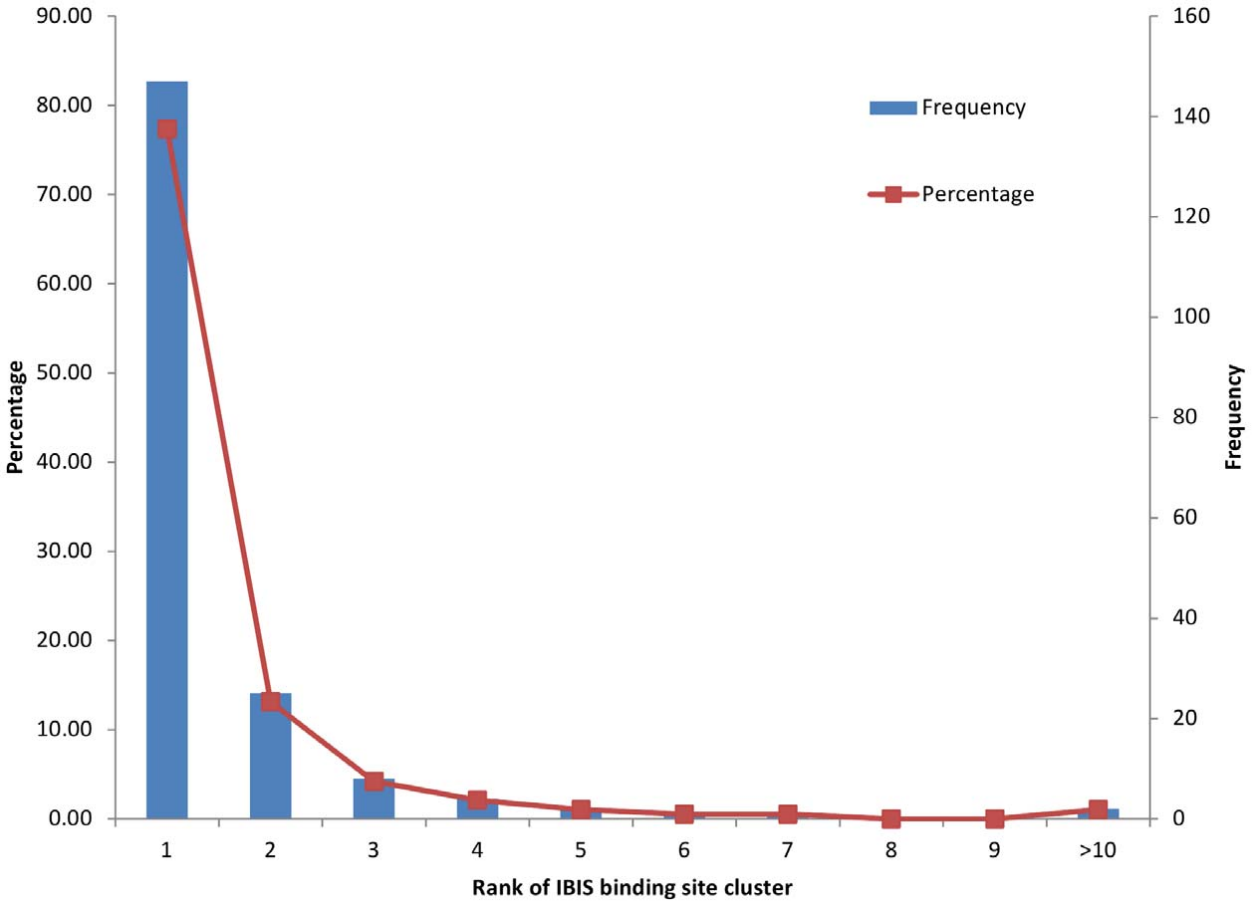
Percentage and frequency of CDD annotated binding sites predicted by IBIS at a given rank.

The Conserved Domain Database (CDD) [26] is a curated collection of ancient families of protein domains along with the manual annotations of functional sites. These functional site annotations have been extracted from the literature or derived by expert manual curation of multiple structure/sequence alignments of family members and can be considered as a standard of truth. First we found 3431 CDD domains with at least one observed IBIS protein-protein binding site. However, only 25% (855) of these CDD domains have manually curated protein-protein binding sites and the remaining 75% of 3431 CDD families are currently missing protein interaction annotations which could be completed using IBIS. Next we used the CDD site annotations as true positives to evaluate IBIS performance. We selected 581 non-redundant PDB chains (chains with 25% identity or higher were removed) out of 3756 chains with available protein-protein CDD binding site annotations from the CDD release 2.16, out of which 278 domains had at least one IBIS binding site cluster (see Methods). We found 231 out of 278 families (83%) where IBIS predicted a binding site which overlapped more than 50% with the CDD binding site annotations, among them 77% were predicted at the top rank (Figure 3).

#### Identifying crystal packing interactions and estimating the false positive rate

IBIS annotation of biologically relevant protein-protein binding sites on a query relies on the conservation of binding sites among homologs. Crystal packing interactions tend not to be found in conserved binding site clusters because the latter are most likely to be biological binding sites. We have evaluated the efficiency of IBIS to correctly identify non-biological crystal packing interactions (as true negatives) using a set of 76 known crystal-packing interactions published previously [27]. We measured the number of false positives, namely, how many times a given pair of chains from the crystal packing set was predicted by IBIS to interact (as a part of a conserved binding site cluster).

As shown in Table 1, out of 76 crystal packing interactions only 8 were present in conserved clusters. Since these crystal packing interactions represent cases of true negatives, we can estimate the fraction of false positives or specificity using this set (see Methods), which in our case turned out to be about 89%. Our results show that the IBIS annotation scheme which groups together valid interactions observed in multiple non-redundant structures can correctly distinguish biological from crystal packing interactions. This observation is in line with the previous studies [24], [28].

**Table 1:** Specificity and sensitivity of IBIS to predict protein-protein interaction.

	Predicted interactions from conserved binding site clusters	Predicted interactions from all binding site clusters
Specificity (76 crystal packinginterfaces)	8 (89%)	25 (67%)^1^
Sensitivity (74 biological interfaces)	50 (68%)^2^	65 (88%)

1 Specificity drops to 67% when interactions from singleton clusters are also considered.

2 Sensitivity drops to 68% when interactions from only conserved clusters are considered.

#### Identifying biological interfaces and estimating the sensitivity

We also evaluated the ability of IBIS to identify biologically relevant PPIs using a set of known protein interactions, in total, 74 biological interactions between protein chains compiled from two previous studies [29], [30]. We found that out of 74 biological interactions 50 of them were identified by IBIS based on recurrence (they were part of conserved binding site clusters) and 15 biological interactions were present in singleton IBIS clusters. Therefore, IBIS yielded a sensitivity of 88% which dropped to 68% if we do not consider singleton binding sites (Table 1).

#### Comparison of IBIS performance with other methods

Finally, based on the test set and results presented by Zhang et al [31] we compared IBIS to recently developed interface prediction methods. The method most similar to ours in terms of ideology, PredUs, was shown previously to outperform cons-PPISP [32], PINUP [33], and ProMate [34] methods and reached 44% precision and 46% recall. Using the same test set we also compared IBIS with a recently developed method called HomPPI, which utilizes sequence homology to infer interaction partners and binding sites [35] (Table 2). It should be noted that out of 188 chains only 171 chains could be employed for the direct comparison with IBIS (due to a number of reasons such as different domain definitions, contact radii, and others) and for additional 25 cases there were no homologous structural complexes above the 30% identity cutoff. For these cases we considered the number of correctly predicted binding site residues to be zero penalizing the estimated IBIS accuracy even though by definition IBIS could not provide predictions for these cases. Since many prediction methods use a non-redundant set of homologs, to speed up the search process we examined IBIS performance after removing redundant homologous structures (with more than 90% identity) from the binding site clusters. As a result, recall dropped dramatically from 72% to 43% (Table 2). It should be mentioned that different methods use different definitions of interactions and non-redundant thresholds which makes it difficult to compare them directly.

**Table 2:** Comparison of IBIS with other protein-protein interaction prediction methods.

Method	Chains	N_p_	N_c_	N_t_	Precision avg (%)	Recall avg (%)
**IBIS**	146	4489	3133	4348	69.7	72.0
**IBIS-NR**	146	2676	1873	4348	72.7	43.0
**HomPPI**	145	4271	2683	5319	62.8	50.4

Here N_p_ and N_c_ represent the number of total and correctly predicted binding site residues respectively. N_t_ is the number of true binding site residues. HomPPI was queried using the test set of 188 chains. Note that IBIS was able to make predictions for only 146 chains, as for the remaining 25 cases there were no homologous structural complexes above the 30% identity cutoff. For these 25 cases we considered the number of correctly predicted binding site residues to be zero penalizing the estimated IBIS accuracy even though by definition IBIS could not provide predictions for these cases.

### Prediction of binding sites between Fe-protein and MoFe-protein from Clostridium pasteurianum nitrogenase complex

The nitrogenase enzyme system catalyzes the nitrogen fixation reaction present in many free-living bacteria, it is composed of two components, molybdenum-iron (MoFe) protein and iron (Fe) protein. MoFe-protein is a hetero-tetramer (α2β2 subunit) with two copies of the FeMo cofactor [36], [37] and two copies of the P-cluster pair, described as containing two Fe_4_S_4_ clusters coupled by two bridging cysteine thiols. [38]. Fe-protein is a homodimer containing a 4Fe-4S cluster and an ATP binding site at the subunit interface. It transfers electrons to MoFe-protein in an ATP-dependent manner. Although both components are very well conserved in terms of their physicochemical properties and overall 3D structure across many different nitrogen fixing bacteria, the nitrogenase enzyme complex from *C. pasteurianum* is quite different from that found in other bacteria.

**Figure 4:**
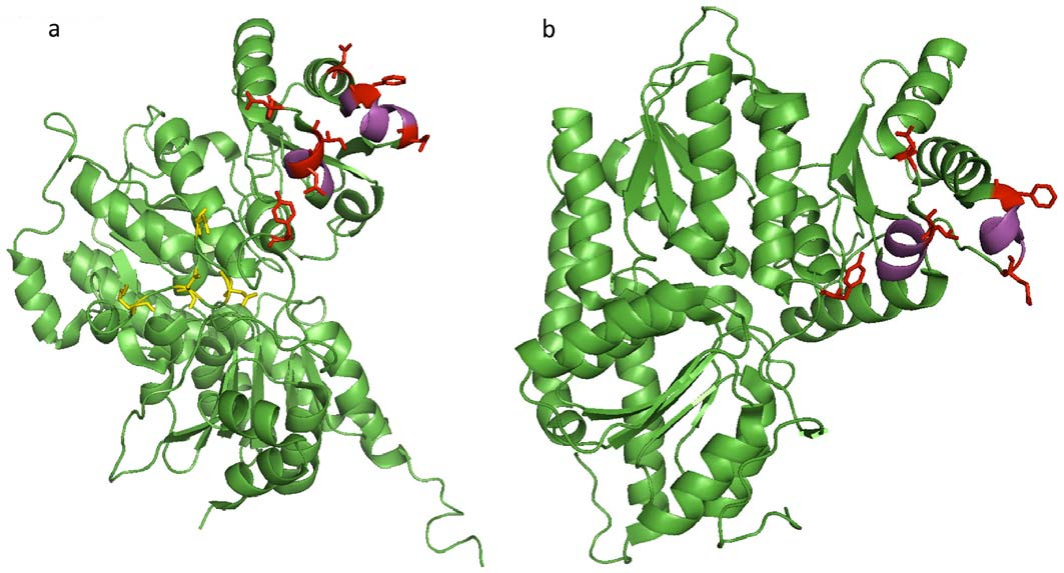
Binding sites between Fe-protein and MoFe-protein. Binding sites inferred on α (a) and β (b) subunits of MoFe protein, PDB chain 1MIO_A and 1MIO_B respectively. Two helical regions assumed to be critical for interaction are shown in magenta. Binding site residues are shown by side chains (in red color) and match with residues predicted by Kim at al. Binding site residues shown in yellow on α subunit (1MIO_A) are part of inserted 50 residues sequence and are not predicted by IBIS.

The MoFe-protein from *C. pasteurianum* has been crystallized as an α2β2 tetramer without the Fe-protein dimer (PDB 1MIO) [39] and currently there is no structure available in the PDB database with the complete nitrogenase complex. In this study we used the β subunit (chain B, 1MIO) of Cp1 protein as an IBIS query to predict putative binding sites of Fe-protein on *C. pasteurianum* MoFe-protein (Cp1) protein based on solved nitrogenase complexes from other bacterial systems. Similarly the α subunit (chain A, 1MIO) was queried in IBIS. We compared inferred sites with MoFe protein and Fe-protein binding sites obtained by docking from previous studies [39], [40]. The sites predicted by IBIS matched quite well (80% of residues) with the docking model, including some key residues, for example, helical regions (residues from 73 to 78 and from 106 to 111) on the β subunit (Figure 4). It is also worth mentioning that IBIS did not predict residues Lys385, Asp387, Asp389 and Asn392 on the α subunit (shown in blue color, Figure 4) which were predicted by Kim et al to be directly involved in recognition of Fe-protein. This is not surprising because the α subunit of MoFe-protein from *C. pasteurianum* (Cp1) has a unique insertion of 50 residues in length and none of the homologs of Cp1 used to infer binding sites contained this insertion.

## Discussion

The coverage and reliability of experimentally determined protein-protein interactions remains quite limited especially for higher organisms, therefore it is important to determine how many of these interactions can be recovered and verified by computational approaches using available information accumulated for protein structural complexes. We present a method which is based on the assumption that the structural location and sequence patterns of protein-protein binding sites are conserved between close homologs. Even though functional annotation transferred from one homologous protein to another can result in incorrect functional assignment, and inference of protein binding interfaces is reliable only among close homologs, we showed that inferring protein binding sites from homologous complexes is a remarkable help to annotate protein binding sites for many unknown protein complexes. Indeed, more than 37000 protein domains are currently annotated with IBIS inferred protein-protein interactions (not counting observed interactions) showing an increase by almost 10000 domains since January 2010. Moreover the IBIS framework can be used to guide and complete the CDD binding site annotations, as we show currently 75% of CDD families are missing protein-protein binding sites which are in turn are present in the IBIS database. We also found that there exists a trade-off between specificity and sensitivity if we use only conserved binding site clusters or clusters supported by only one observation (singletons). It implies that different strategies of inference should be applied depending on a particular task of prediction.

There are a few inference based approaches to predict protein-protein binding sites [5]–[8], [31], [35]. For example, PredUs, uses the data independent of any homology assignments trying to maximize the coverage of an entire protein-protein interaction network, while IBIS operates on the level of close homologs and pays particular attention to verify the evidence which the prediction is based on. Interestingly we showed that a considerable increase in IBIS accuracy is observed if all available data on structures of homologous complexes is used compared to the approach where only a non-redundant set of complexes is employed. Even identical or very similar proteins may differ somewhat in binding site locations due to their dynamical and allosteric properties. Moreover proteins with the same overall topology might form different oligomeric states and have peculiar structural or sequence features which might be responsible for their specific binding properties required for function or adaptation to various environments [41]–[43]. Here we show that all structural data represents an invaluable source of information on binding site annotation and allows for an easier interpretation of the results.

Knowledge of the true oligomeric assembly/state of a protein is critical for correct annotation of functional binding sites. Deciphering the correct state is tedious and experimental methods like analytical ultracentrifugation, gel filtration, mass spectrometry and others provide useful but still limited information on the biologically relevant assembly. The only way to study the atomic details of protein-protein interactions is to use structures present in the Protein Data Bank (PDB) [44]. The information about biological units in the PDB ASU can be inconsistent and represents a source of error in annotating protein-protein interactions. Indeed, nowadays proteins sometimes are being crystallized without the extensive biochemical or biophysical characterization of their oligomeric states. Different computational methods have been proposed in this respect to identify the biological oligomeric complexes but only a few of them may decipher biological assemblies from crystalline states with high enough accuracy [45]–[48]. Such methods reconstruct both biological and crystal-packing interfaces by applying crystallographic symmetry operations, then differentiate the biological from the crystal-packing interfaces by computational criteria. In our work we showed that IBIS can handle both of these tasks. About 90% of crystal packing interactions can be correctly identified by the IBIS algorithm, which employs information on evolutionary conservation of protein-protein binding sites. At the same time about 45% of biological interfaces that are not present in the PDB asymmetric unit can be reconstructed by IBIS without the prior knowledge of crystal parameters of the query protein. The other 55% of valid interfaces might either represent the interfaces specific for a certain protein subfamily or be present only in remotely related proteins and therefore cannot be derived reliably using conserved homologs. The uncovered interfaces can be used as a guide in selecting the new protein complex targets in protein structural genomics.

## Supporting Information

Figure S1 **Distribution of oligomeric states of multimeric structures with observed interactions in IBIS.** State “−1” correspond to structures that either could not be processed by PISA or no stable assembly was predicted by PISA.
(TIF)

Figure S2 **Distribution of the number of chains for structures predicted as monomers by PISA but present as multimers in PDB ASU.** Bin “1” corresponds to intra-chain domain-domain interactions. Other structures represent cases with potential crystal packing interactions.
(TIF)

Figure S3 **Distribution of PISA predicted multimeric states for structures present as a single chain in PDB ASU.**
(TIF)
