# Supplementary figures and images for: Homology Inference of Protein-Protein Interactions via Conserved Binding Sites
Source: PLoS One. 2012 Jan 31;7(1):e28896. doi: 10.1371/journal.pone.0028896 (PMC3269416; doi:10.1371/journal.pone.0028896)

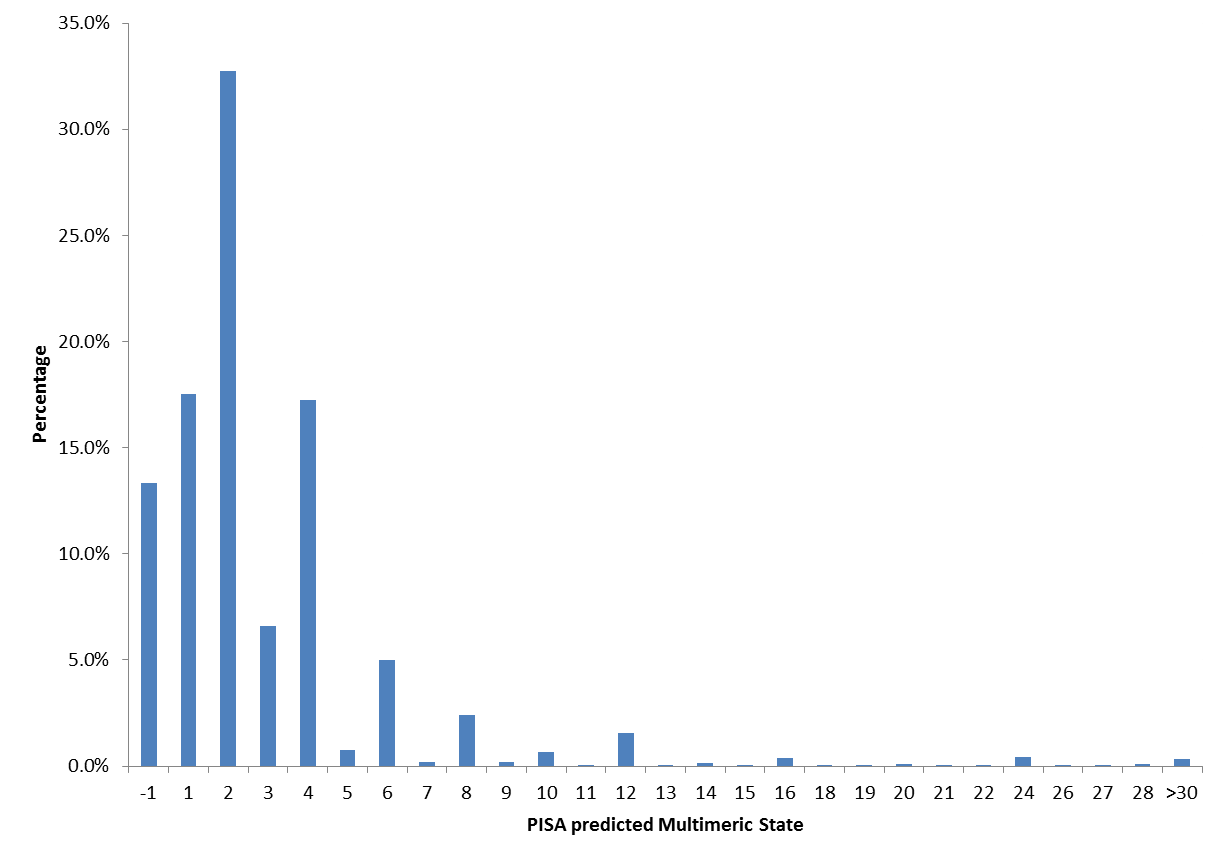

Supplement: Figure S1 — Distribution of oligomeric states of multimeric structures with observed interactions in IBIS. State “−1” correspond to structures that either could not be processed by PISA or no stable assembly was predicted by PISA. (TIF) [file pone.0028896.s001.tif]

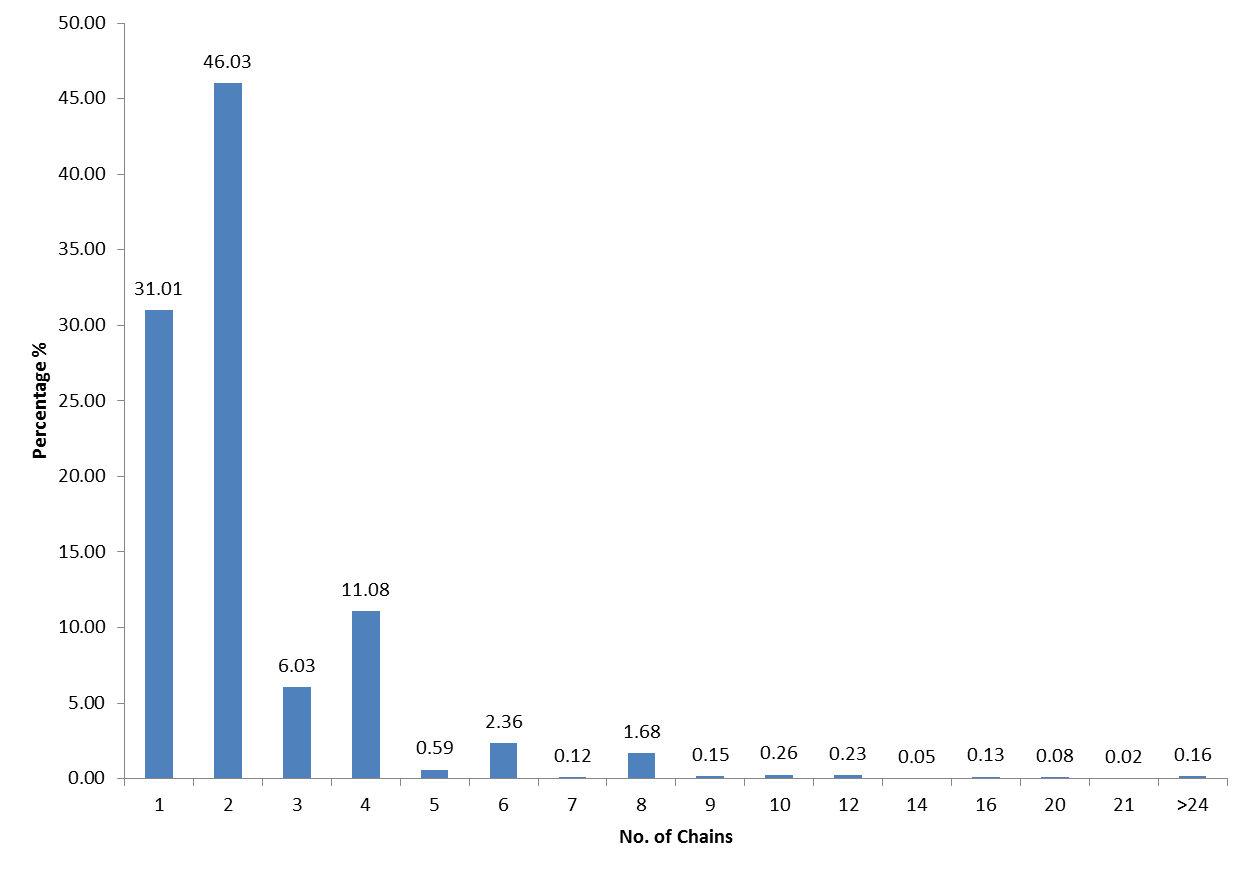

Supplement: Figure S2 — Distribution of the number of chains for structures predicted as monomers by PISA but present as multimers in PDB ASU. Bin “1” corresponds to intra-chain domain-domain interactions. Other structures represent cases with potential crystal packing interactions. (TIF) [file pone.0028896.s002.tif]

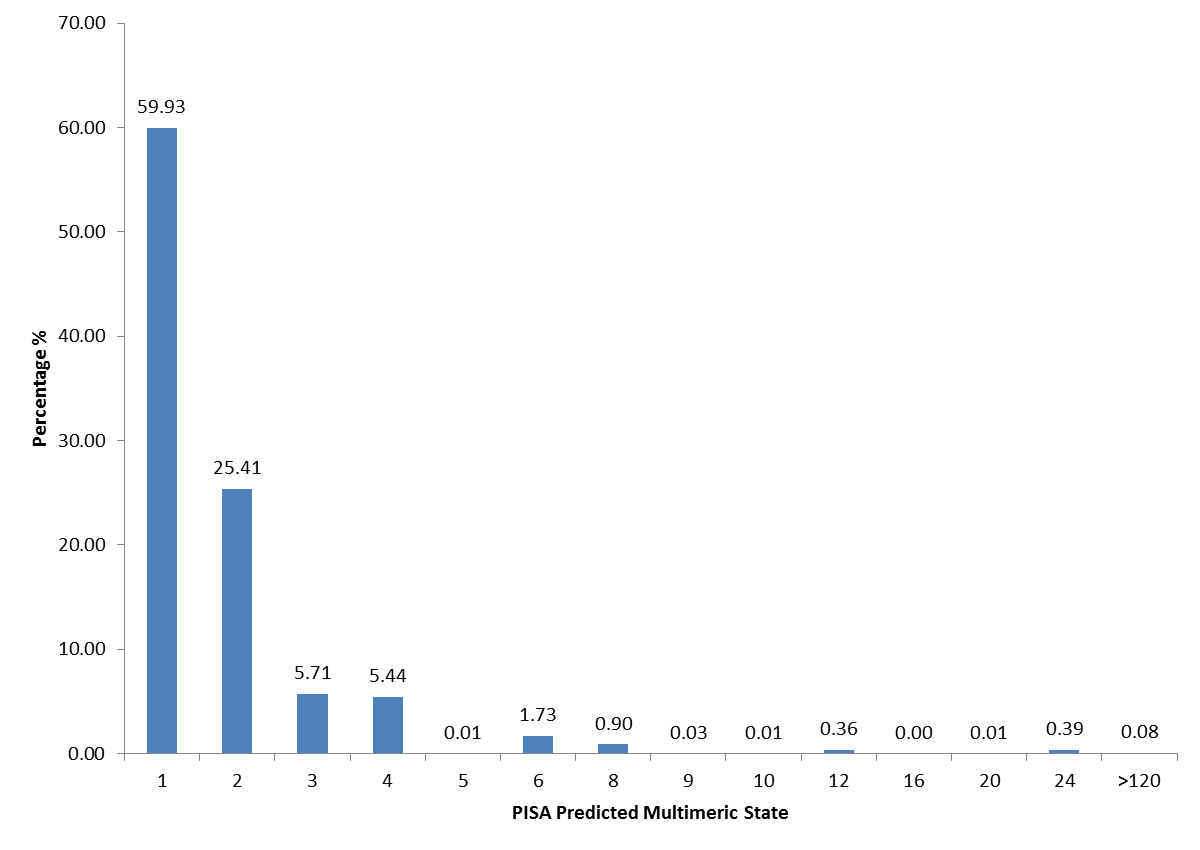

Supplement: Figure S3 — Distribution of PISA predicted multimeric states for structures present as a single chain in PDB ASU. (TIF) [file pone.0028896.s003.tif]
